# Additional obstacles in carbon nanotube growth by gas-flow directed chemical vapour deposition unveiled through improving growth density†

**DOI:** 10.1039/c9na00209j

**Published:** 2019-09-02

**Authors:** Takashi Tsuji, Kenji Hata, Don N. Futaba, Shunsuke Sakurai

**Affiliations:** CNT-Application Research Centre, National Institute of Industrial Science and Technology (AIST) Central 5, 1-1-1, Higashi Tsukuba Ibaraki 305-8565 Japan d-futaba@aist.go.jp shunsuke-sakurai@aist.go.jp

## Abstract

Here, we demonstrate an approach of increasing the density of ultralong carbon nanotube (CNT) growth by combining a fast-heating method developed by Huang *et al.* (*J. Am. Chem. Soc.*, 2003, **125**, 5636–5637) with catalyst support engineering. Specifically, using graphene oxide as a catalyst support for iron oxide (Fe_3_O_4_) catalyst nanoparticles, we achieved high density growth of CNTs grown by the “kite-mechanism”. Our analysis revealed that the fast-heating method reduced undesired aggregation of the catalyst nanoparticles, which has been reported to be a primary limitation mechanism, by shortening the time between substrate heating and CNT growth. In addition, the use of the graphene oxide support led to controllable and uniform dispersion of catalyst nanoparticles in relatively high density which provided increased process control by extending the time before the onset of catalyst aggregation. Together, these approaches suppressed the aggregation of the catalyst nanoparticles, which facilitated the “tip-growth” mode instead of the “root-growth” mode, and led to the high density growth of ultralong CNTs. Our results also indicate additional limitations and complexities on the high density CNT growth by the kite-growth approach, which limit high density synthesis.

## Abstract 2

We report growth of ultralong carbon nanotubes by combining a fast-heating chemical vapour deposition process with catalyst support engineering.

## Graphical abstract

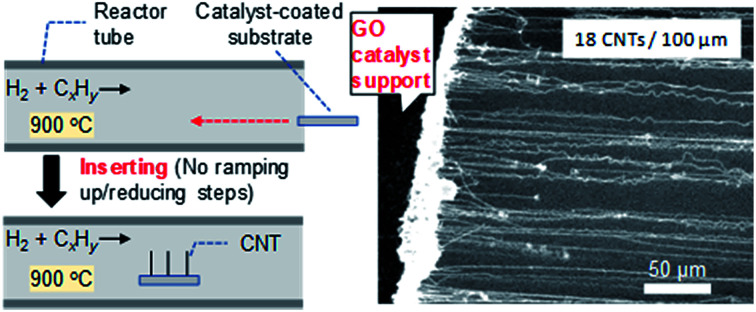

## Introduction

Gas-flow directed chemical vapour deposition (CVD) is a process to grow ultralong and highly crystalline carbon nanotubes (CNTs). In this process, the growth of a nanotube proceeds with one end of the CNT anchored to the substrate and the other end of the CNT, which contains the catalyst nanoparticle, floating off from the substrate and continuing to grow along the gas-flow direction. The CNT is supported by buoyant forces from the convective gas flow produced by the temperature gradient between the flowing gas and the substrate. This process is referred to as the “kite-mechanism” or “tip-growth”.^1–5^ Growth of CNTs in this manner has shown the capability to grow ultralong CNTs up to 55 cm and they possess exceptionally high mechanical strength due to their defect free structure.^6^ In fact, the tensile strength of the bundle made of these CNTs has recently been reported to reach 80 GPa,^7^ and these CNTs have exhibited excellent electrical properties, such as long mean free path and high mobility.^8^ These outstanding properties demonstrate the benefits of such a growth process.

Although much progress has been made on growing ultralong CNTs by this gas-flow directed CVD, increasing the growth efficiency in terms of CNT number density remains a challenge. The typical density of the ultralong CNTs is only several CNTs/100 μm where only a few CNTs are successful in growing ultralong across a 100 μm wide region of catalyst nanoparticles, and the highest reported density was 60 CNTs/100 μm by Liu *et al.*^9^ This is in stark contrast to highly dense (∼10^3^–10^4^ CNTs only in the 1 μm^2^ catalyst region) CNTs grown from substrates based on the “root-growth” or “base-growth” mechanism which have shown catalyst activities as high as 84%.^10^

One of the primary causes of low CNT density, *i.e.* catalyst activity, in kite-growth is thought to be the formation of aggregated large catalyst nanoparticles. Since large nanoparticles tend to grow CNTs with the catalysts “rooted” to the surface,^11^ the mobility of particles on the catalyst support accelerated at the high process temperature can disable kite-growth. The previously reported process of gas-flow directed CVD consisted of (1) catalyst deposition on a substrate (usually SiO_2_), (2) reduction of the catalyst nanoparticles (usually by heating in a hydrogen (H_2_) environment for a few minutes), and finally (3) CNT growth. The highest values of CNT number density were obtained by optimization of the above process. Specifically, Liu *et al.* reported 60 CNTs/100 μm by optimizing the catalyst deposition method (post-annealing of Fe after deposition),^9^ and Zhou *et al.* obtained 20–30 CNTs/100 μm by optimizing a copper catalyst.^12^ However, it is fundamentally difficult to avoid catalyst nanoparticle aggregation in the above process because of the high mobility of metal catalyst nanoparticles on SiO_2_ surfaces at a high temperature range.

Catalyst support engineering and the fast-heating process are two approaches to overcome the above problem in the previous research. Since the interaction between the catalyst and the catalyst support layer plays an important role in the stability of catalyst nanoparticle arrays for root-growth systems,^13–16^ a number of approaches have similarly employed “catalyst support engineering” on kite-growth. For example, the use of silica nanospheres^17^ or graphene/graphite sheets^18^ has shown suppression of catalyst aggregation, which resulted in improved number density of ultralong CNTs compared to the commonly used SiO_2_ support (∼3 CNTs/100 μm^17^), but not exceeding the highest value of kite-growth. The fast-heating method, developed by Huang *et al.*,^1,19^ is one of the methods particularly suitable for gas-flow directed CNT growth. In this method, the catalyst-coated substrate is rapidly transferred into a preheated reactor already set at the CNT growth environment. As a result, the exposure time of catalyst particles to a high temperature environment is drastically shortened, thus reducing the probability of catalyst aggregation. Huang *et al.* grew ultralong CNTs by using several varieties of catalysts, such as iron/molybdenum (Fe/Mo) nanoparticles, iron/platinum nanoparticles, Fe/Mo molecular clusters and carbon sources, such as carbon monoxide (CO), methane (CH_4_) or methanol.^1^ Although the number density of the ultralong CNT was not mentioned in the report, estimation of the CNT density from the published images appears to be below 1 CNT/100 μm in most cases with a maximum of 7–8 CNTs/100 μm (in the case of the Fe/Mo catalyst and CO carbon source). Taken together, these results indicate that the use of the fast-heating method alone or catalyst support engineering alone is not sufficient to grow high density CNTs.

In this study, we have combined the use of the fast-heating approach with catalyst support engineering as a method to increase CNT number density. Specifically, using iron oxide (Fe_3_O_4_) nanoparticles cast with graphene oxide (GO) support as catalysts, the density of ultralong CNTs grown by the fast-heating method reached ∼20 CNTs/100 μm. While not the highest reported, this value compares well with that reported by Liu *et al.*^9^ and Zhou *et al.*^12^ The graphene oxide allowed for well-separated catalyst nanoparticles across the surface and the fast-heating process facilitated the CNT growth before significant catalyst aggregation occurred. Combination of these effects led to the high density growth of ultralong CNTs. Our results further show that the achievement of high density CNT growth by a kite-mechanism is highly complex and only begins with the preparation of suitably spaced and active nanoparticles, followed by a suppression in the interaction between growing nanotubes.

## Results and discussion

**Fig. 1:**
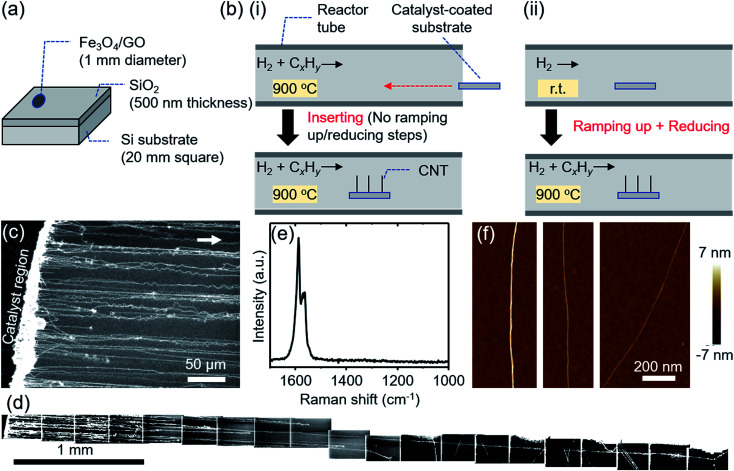
(a) Schematic illustration of the catalyst-coated substrate. (b) Schematic illustration showing the synthesis process of (i) fast-heating and (ii) normal-heating methods. r.t. represents room temperature. (c–f) Characterization of ultralong CNTs grown under typical fast-heating conditions, where CH_4_ was flowing with no delay time. (c) SEM image of ultralong CNTs grown from the catalyst region (Fe_3_O_4_/GO film). The arrow indicates the gas flow direction. (d) Overall SEM images of an ultralong CNT. (e) Raman spectrum of the CNTs. (f) AFM images of the CNTs.

To begin, we demonstrate high density growth of ultralong CNTs by using a mixed catalyst system of Fe_3_O_4_ nanoparticles and GO flakes (Fe_3_O_4_/GO) and a fast heating method. A catalyst region composed of Fe_3_O_4_/GO films (diameter: ∼1 mm) was prepared by drop-casting an aqueous dispersed solution of Fe_3_O_4_ nanoparticles and GO flakes onto a SiO_2_/Si substrate (Fig. 1a). The catalyst-coated substrate was shuttled directly into the heated growth environment. Specifically, the sample was transferred into the preheated furnace reactor while CH_4_ and H_2_ gases were already flowing (Fig. 1b(i)). Therefore, in stark contrast to previously reported synthesis processes (Fig. 1b(ii)), we did not employ an independent reduction step prior to the growth step. CNTs grew from the catalyst region on the SiO_2_/Si substrate along the gas-flow direction (Fig. 1c). We chose to use GO flakes to create a system of well-separated catalyst nanoparticles, whose spacing is larger than that cast on a substrate without GO. The functional groups, defects or/and edges of GO acted as pinning sites for the catalyst nanoparticles and therefore aided in dispersion of the catalyst nanoparticles on the GO support. Characterization of the GO flakes used in this study is supplemented in Fig. S1.† The CNT number density was estimated to be 18 CNTs/100 μm from scanning electron microscopy (SEM) images (Fig. 1c). Tracing CNTs by SEM revealed that the length of the CNTs was up to 5.7 mm (Fig. 1d). CNTs grew across the deep trenches as reported by previous reports,^1,2,20^ which is indicative of the gas-flow directed growth (Fig. S2†). Raman spectroscopy of the grown CNTs suspended above trenches showed a strong graphitic (G-) band, while the disorder (D-) band was not detectable around ∼1350 cm^−1^, indicating that the grown CNTs possessed high crystallinity (Fig. 1e). The typical diameter of the CNTs characterized from height profiles of atomic force microscopy (AFM) images was 2–6 nm (Fig. 1f).

**Fig. 2:**
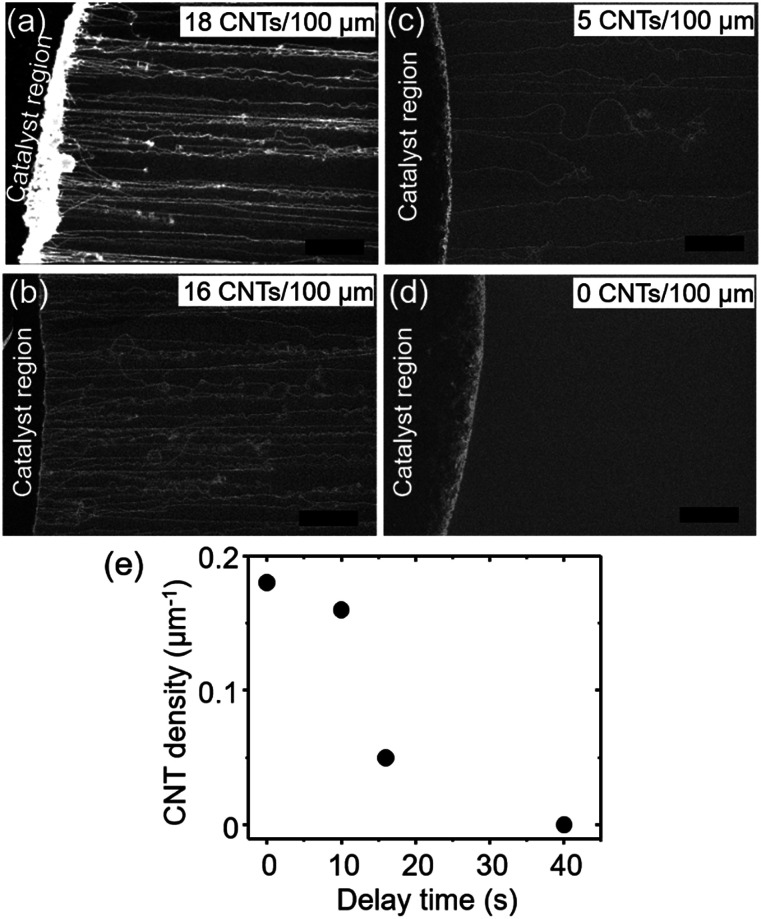
(a–d) SEM images of the CNTs grown after the H_2_ reduction process at different times; (a) 0 s, (b) 10 s, (c) 16 s, and (d) 40 s (scale bars: 50 μm). (e) Number density of CNTs as a function of the delay time, interval time between CH_4_ supply and the insertion of the catalyst substrate into the centre of the furnace.

In order to understand the importance of the fast-heating method, we examined the effect of the timing of the carbon source delivery on the CNT number density. Specifically, we modulated the “delay time” between the moment the sample reached the center of the furnace and the time the gas flow, containing carbon source, reached the sample position (see ESI 3† for a detailed definition). Number densities of CNTs estimated by SEM images (Fig. 2a–d) were plotted as a function of this “delay time” (Fig. 2e). As a whole, the trend of the CNT densities resembled an inverse S-curve shape characterized by (1) a region of high CNT density, (2) a region of decreasing CNT density, and (3) a region of no CNT growth. In the high CNT density region (<10 s), the numbers of observed CNTs were found to be the highest (Fig. 2a). The second region (beginning at ∼10 s) was characterized by a sharp decrease in the CNT density. Finally, in the no CNT growth region, CNT number density approached zero. Importantly, the window of the high CNT density region (∼10 s) was estimated to be comparable to the heating time for the catalyst to reach the reactor temperature (900 °C). When a thermocouple probe was inserted into the furnace at a speed similar to that of the actual substrate (∼10 mm s^−1^) as a simple test, a further ∼15 s was required for the probe temperature to reach 900 °C from ∼700 °C, the value when the probe reached to the center of the furnace. The result clearly shows the necessity of the catalyst nanoparticles to react with the carbon source and start the CNT growth within a particularly short time after the substrate/catalyst reaches growth temperature for achieving a high density growth of the ultralong CNTs.

**Fig. 3:**
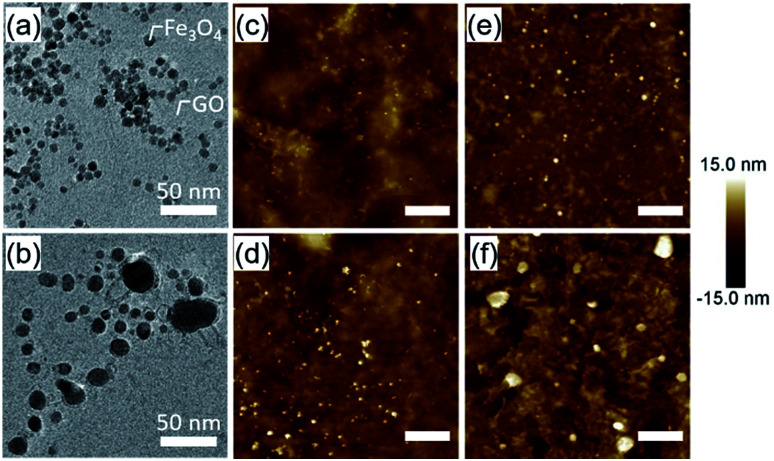
Catalyst evolution of the Fe_3_O_4_/GO system. (a and b) TEM images (a) before and (b) after 6 s annealing at 900 °C in H_2_. (c–f) AFM images (c) before and (d–f) after annealing at 900 °C in H_2_ for (d) 6 s, (e) 30 s, and (f) 180 s (scale bars: 200 nm).

We used transmission electron microscopy (TEM) and AFM to investigate the evolution of the catalyst nanoparticles in the CVD process environment. A silicon nitride membrane grid with micropores was immersed into the aqueous solution of 5 nm Fe_3_O_4_ nanoparticles and the GO for 10 min. ∼5 nm particles supported on the GO layers covering the microporous region were observed (Fig. 3a and S3†). The TEM grid was then transferred to the CVD reactor preheated at 900 °C and kept only for 6 s in H_2_ using the fast-heating method. TEM observation showed a reduction in catalyst number density and an increase in size (∼13 nm on average) by this fast-heating (Fig. 3b). Similarly, a Fe_3_O_4_/GO catalyst-coated SiO_2_/Si substrate was also heated at 900 °C in a H_2_ environment for a set time (6, 30, and 180 s) and observed by AFM. AFM observation (Fig. 3c–f) also showed the increase of particle size with heating time. These results further indicate the relatively high rate at which the catalyst nanoparticles coarsen by enhanced particle movement on the GO support due to the high process temperature, which is in agreement with the CNT density delay time results of Fig. 2. Importantly, these results clearly show the importance of the fast-heating method for suppressing catalyst aggregation during the H_2_ reduction.

**Fig. 4:**
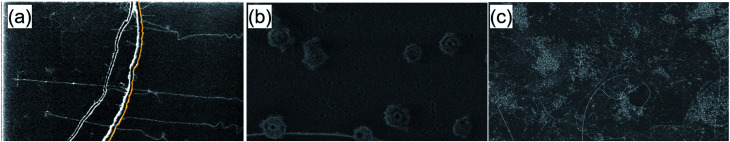
(a) CNTs grown using the Fe_3_O_4_ catalyst without GO on SiO_2_/Si. The area to the left side of the yellow line is a catalyst region. (b and c) SEM images of the catalyst region (b) without and (c) with the GO support after the growth process.

To demonstrate the importance of using the GO support, the Fe_3_O_4_ nanoparticle catalyst, alone, was deposited without using the GO support, and then subjected to the fast heating CVD process. Significantly decreased CNT number density (∼2 CNTs/100 μm) was observed in this case (Fig. 4a). This result indicates the critical role of the GO support in increasing the number density of grown CNTs by nearly 10-times (∼20 CNTs/100 μm) as shown in Fig. 1c likely by forming a well-separated array of Fe_3_O_4_ nanoparticles.

To investigate the role of GO in the separation of catalyst nanoparticles, a comparative SEM observation of the catalyst systems with and without GO was performed after the growth process. In the case where the Fe_3_O_4_ catalyst, alone, was deposited onto the SiO_2_/Si substrate, extremely coarsened aggregates with a size of ∼1 μm were observed in large numbers (Fig. 4b). In stark contrast, the SEM image of the Fe_3_O_4_/GO system showed no large micrometre-scale particles, but rather a large number of smaller particles and many entangled CNTs (Fig. 4c). Taken together, these results provide clear evidence of the ability of the GO support for a well-separated Fe nanoparticle array after coating and even after heating up to the CNT growth environment.

As mentioned previously, large nanoparticles formed by aggregation tend to grow CNTs by a “root-growth” mode instead of a “tip-growth” mode, resulting in short MWCNTs. Both the fast-heating method and the use of the GO support facilitated the CNT growth before substantial catalyst coarsening could occur. Our results showed that the fast-heating method shortened the necessary catalyst reduction time prior to the growth step, thus reducing the time for catalyst aggregation to occur. In addition, use of GO as the catalyst support improved the dispersibility of the catalyst nanoparticles, thus increasing our process control by extending the allowable window of time between substrate insertion and carbon feedstock contact. In doing so, the initiation of CNT nucleation before catalyst aggregation improved the number density of the ultralong CNTs. It should be noted that direct comparison using neither the fast-heating method nor GO support resulted in no observable CNT growth under our experimental conditions (Fig. S4†). This result shows the significance of the collective effects of the fast-heating method and the use of GO supports.

**Fig. 5:**
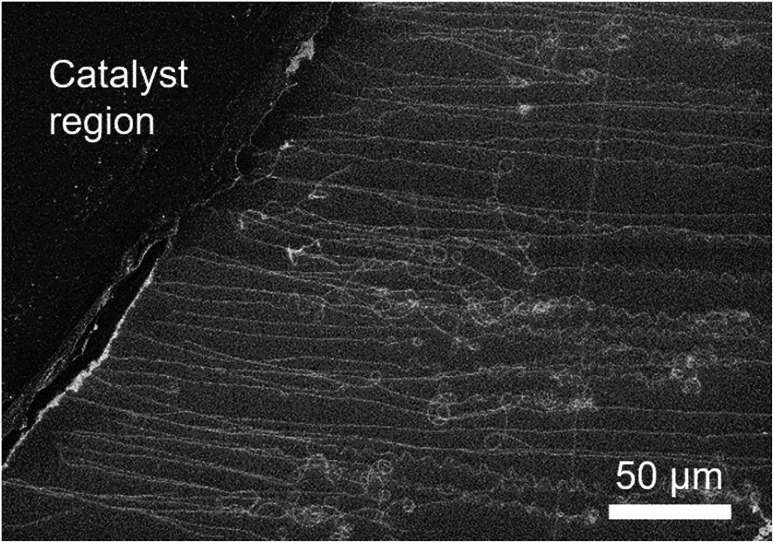
SEM image of CNTs grown from the catalyst region composed of Fe_3_O_4_ and e-DIPS CNTs.

Finally, we recognize that despite our approach of using a fast-heating method and catalyst engineering (such as the GO catalyst support) to increase the obtained CNT densities, we were unsuccessful in achieving higher densities than previously reported ones. Specifically, modulation of the Fe_3_O_4_ concentration did not show any significant changes in the grown CNT density (10–20 CNTs/100 μm, Fig. S5†). We believe our results to be general to other catalyst systems as similar results were obtained using a catalyst support composed of an aggregate of the as-grown CNTs (e-DIPS CNTs). The observed CNT number density was estimated to be 22 CNTs/100 μm (Fig. 5). Importantly, despite the extreme structural difference in these two catalyst/support systems, the resulting number density of grown CNTs converged to the similar values of ∼20 CNTs/100 μm.

The above results suggest that the number of ultralong CNTs, in our experiments, is not limited by the number of catalysts which nucleate the tip-growth, which indicates that increasing the density of flying CNTs represents a more complex process which extends beyond the suppression of catalyst aggregation and catalyst–support interaction. Analogous to the formation of a CNT forest, which requires a combination of a growth environment supporting a high catalyst activity,^10^ appropriately spaced catalyst nanoparticles based on their size,^21^ and stable catalyst array^13^ to ensure vertical growth, the achievement of kite-growth CNTs also requires a strict set of conditions. Further, the interaction between CNTs adds to the complexity. Therefore, from our results, we propose that the following steps are additionally necessary for high density kite-growth CNTs in a single growth: (1) liftoff and (2) sustained buoyancy for extended lengths.

First, liftoff is defined as the moment the catalysts leave the substrate surface and begins the kite-growth process. This step is particularly complex as the growth directionality in the early stages of growth is largely random as observed (Fig. 4c), which results in collisions and entanglements among neighboring CNTs. Our observations strongly indicate that this is another severe bottleneck in achieving high density, which would require either large catalyst spacing or unidirectional growth. Second, sustaining the buoyancy for extended growth represents another obstacle for high density growth. In short, entanglements between highly-dense CNTs in the gas flow will act to disrupt the buoyant forces necessary for kite-growth. In experiments presented here, we observed many entangled CNTs in our samples just outside the catalyst region and after the liftoff step, as shown in Fig. 1c. Therefore, as outlined above, although an array of stable and catalytically active nanoparticles is an important first step to increase the density, limiting intertube entanglements just after liftoff remains a significant obstacle. Resolving the entanglement during and after liftoff remains a significant issue, and we expect that our work will facilitate future work to address these issues.

## Conclusions

In conclusion, we demonstrated an approach of increasing CNT density of “kite-growth” CNTs by combining a fast heating CVD process with catalyst support engineering. Specifically, using GO as a catalyst support for Fe_3_O_4_ catalyst nanoparticles a high density growth of ultralong CNTs could be demonstrated. Microscopic investigation of the fast heating process and catalyst support engineering revealed that the combined usage of these two techniques essentially extended the time between the insertion of the catalyst-coated substrate into the CVD reactor and the start of the growth process. As catalyst aggregation occurs on a fast time-scale, the fast-heating approach afforded a shortened catalyst heating step, and the GO support provides well-separated catalyst nanoparticles more resistant to catalyst aggregation. These collective effects suppress the aggregation of the catalyst nanoparticles and led to the high density growth of ultralong CNTs. Further, we also show that other limiting factors exist in increasing the density of kite-growth CNTs.

## Experimental

For the preparation of standard catalyst systems composed of iron oxide (Fe_3_O_4_) and graphene oxide (GO), first, aqueous dispersions of 5 nm Fe_3_O_4_ nanoparticles (0.4 mg ml^−1^, Aldrich) and GO flakes (4 mg ml^−1^, Aldrich) were mixed in the volume ratio of 1 : 1. For each experiment described in the manuscript, mixing mass ratios of GO and Fe_3_O_4_ were adjusted within the range of 0–10 : 1. Then the mixture (1 μl) was drop-cast onto a SiO_2_/Si substrate (thickness of SiO_2_: 500 nm; size: 20 mm square) and then dried at 200 °C for 2 min. For the preparation of the catalyst system composed of Fe_3_O_4_ and the e-DIPS CNT catalyst support, an agglomerate (∼10 mm in size) of the as-grown e-DIPS CNT (Meijo eDIPS EC2.0, Meijo Nanocarbon) was immersed in a dispersion of Fe_3_O_4_ (0.05 mg ml^−1^, aqueous solution of 50 vol% ethanol) for 2 min. Then, a wet e-DIPS CNT aggregate was taken from the dispersion, transferred onto a SiO_2_/Si substrate, and then dried at 200 °C for 2 min.

Growth of CNTs was performed using an automated 1-inch horizontal quartz tube using the fast-heating CVD process. The substrate put on the quartz holder was transferred into the furnace preheated at 900 °C with a speed of 10 mm s^−1^ in an environment of H_2_ : CH_4_ = 1 : 1 (total flow: 1000 sccm) and then kept for 10 min.

Characterization of the grown CNTs and the catalyst was performed using a SEM (Hitachi, S-4800), AFM (Bruker, Dimension FastScan) and Raman spectrometer (Thermo Fisher Scientific, NICOLET ALMEGA XR) with 532 nm excitation. The Raman spectrum was obtained from the suspended segments of CNTs across a trench in a SiO_2_/Si substrate shown in Fig. S2.† The trenches on silicon wafers were fabricated using a laser with ∼100 μm in width and ∼63 μm in depth. TEM (TOPCON EM-002B) was used to observe Fe_3_O_4_ nanoparticles on GO flakes. A silicon nitride membrane grid with 2 μm micropores (SN100-A20MP2Q05, ALLIANCE Biosystems) was first immersed into an aqueous solution of 0.1 vol% (3-aminopropyl)triethoxysilane (Wako) for 30 min and rinsed with water. After drying (105 °C, 30 min, in air), the grid was immersed into the aqueous solution of 5 nm Fe_3_O_4_ nanoparticles and GO for 10 min. Then the grid was rinsed in pure water then ethanol and finally dried in air (105 °C, 1 hour).

## Conflicts of interest

There are no conflicts to declare.

## Supplementary Material

NA-001-C9NA00209J-s001

## References

[cit1] Huang S., Woodson M., Smalley R., Liu J. (2004). Nano Lett..

[cit2] Jin Z., Chu H., Wang J., Hong J., Tan W., Li Y. (2007). Nano Lett..

[cit3] Wen Q., Zhang R., Qian W., Wang Y., Tan P., Nie J., Wei F. (2010). Chem. Mater..

[cit4] Zhang R., Zhang Y., Wei F. (2017). Chem. Soc. Rev..

[cit5] Wang X., Li Q., Xie J., Jin Z., Wang J., Li Y., Jiang K., Fan S. (2009). Nano Lett..

[cit6] Zhang R., Zhang Y., Zhang Q., Xie H., Qian W., Wei F. (2013). ACS Nano.

[cit7] Bai Y., Zhang R., Ye X., Zhu Z., Xie H., Shen B., Cai D., Liu B., Zhang C., Jia Z., Zhang S., Li X., Wei F. (2018). Nat. Nanotechnol..

[cit8] Li S., Yu Z., Rutherglen C., Burke P. J. (2004). Nano Lett..

[cit9] Liu H., Takagi D., Chiashi S., Homma Y. (2009). Nanotechnology.

[cit10] Futaba D. N., Hata K., Namai T., Yamada T., Mizuno K., Hayamizu Y., Yumura M., Iijima S. (2006). J. Phys. Chem. B.

[cit11] Zhang R., Xie H., Zhang Y., Zhang Q., Jin Y., Li P., Qian W., Wei F. (2013). Carbon.

[cit12] Zhou W., Han Z., Wang J., Zhang Y., Jin Z., Sun X., Zhang Y., Yan C., Li Y. (2006). Nano Lett..

[cit13] Amama P. B., Pint C. L., Kim S. M., McJilton L., Eyink K. G., Stach E. A., Hauge R. H., Maruyama B. (2010). ACS Nano.

[cit14] Chen G., Sakurai S., Yumura M., Hata K., Futaba D. N. (2016). Carbon.

[cit15] Tsuji T., Hata K., Futaba D. N., Sakurai S. (2016). J. Am. Chem. Soc..

[cit16] Chen G., Futaba D. N., Hata K. (2017). MRS Bull..

[cit17] Xie H., Zhang R., Zhang Y., Li P., Jin Y., Wei F. (2013). Carbon.

[cit18] Xie H., Zhang R., Zhang Y., Zhang W., Jian M., Wang C., Wang Q., Wei F. (2014). Chem. Commun..

[cit19] Huang S., Cai X., Liu J. (2003). J. Am. Chem. Soc..

[cit20] An J., Zhan Z., Krishna H., Vijayalal S., Sun G., Hansen R. V., Zheng L. (2015). J. Mater. Chem. C.

[cit21] Chen G., Davis R. C., Futaba D. N., Sakurai S., Kobashi K., Yumura M., Hata K. (2016). Nanoscale.

