# Supplementary material for: Additional obstacles in carbon nanotube growth by gas-flow directed chemical vapour deposition unveiled through improving growth density
Source: Nanoscale Adv. 2019 Sep 2;1(10):4076–81. doi: 10.1039/c9na00209j (PMC9418430; doi:10.1039/c9na00209j)
Supplement: NA-001-C9NA00209J-s001 [file NA-001-C9NA00209J-s001.pdf]

Electronic Supplementary Material (ESI) for Nanoscale Advances.
This journal is © The Royal Society of Chemistry 2019

Electronic Supplementary Information (ESI) for

Additional obstacles in carbon nanotube growth by gas-flow directed chemical vapour deposition unveiled through improving growth density

Takashi Tsuji, Kenji Hata, Don N. Futaba*, and Shunsuke Sakurai*

CNT-Application Research Centre, National Institute of Advanced Industrial Science and Technology, Central 5, 1-1-1 Higashi, Tsukuba, Ibaraki, 305-8565, Japan

*Corresponding author: d-futaba@aist.go.jp, shunsuke-sakurai@aist.go.jp

Supplementary Information 1: Characterization of GO used in this study

Our characterization of the graphene oxide (GO) used in this study indicates that the GO exist as isolated flakes or their aggregates. We performed microscopic (AFM/SEM) and spectroscopic characterization of the graphene flakes to provide the size, shape, and quality of our GO. To characterize the GO, an aqueous dispersion of GO (~0.01 mg/ml) was spin-coated onto a SiO_2/Si substrate. The ~10 µm aggregate of the GO flakes and separated flakes of several µm in size were observed in SEM image (Fig. S1a). The thickness of the GO flakes characterized by AFM images was ~1 nm (Fig. S1b)). Raman spectrum of the GO flakes casted on the SiO_2/Si substrate shows G and D bands at ~1600 and ~1350 cm^{-1}, respectively, with G/D ratio of ~1, which is the typical feature of GO (Fig. S1c)

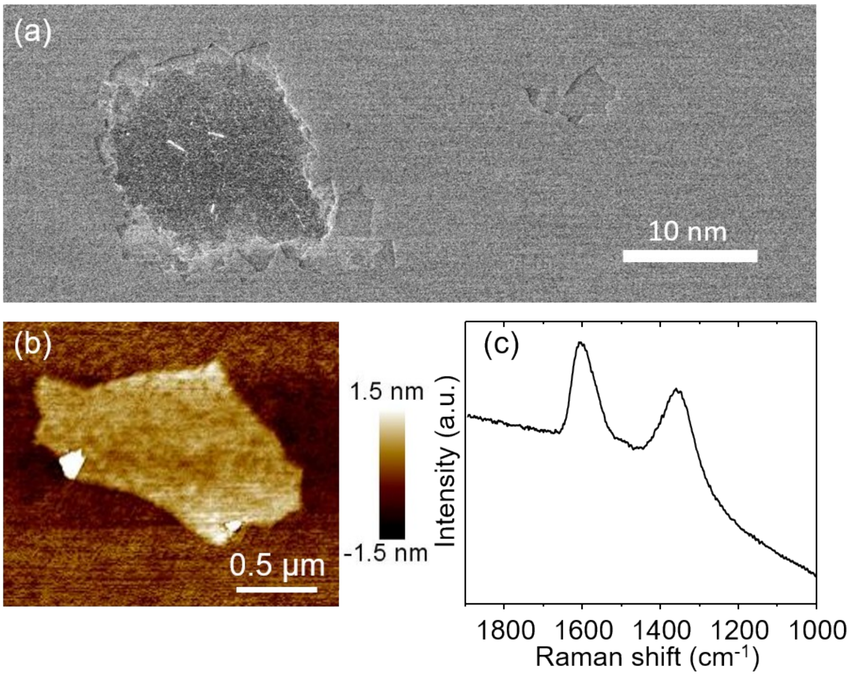

Figure S1. (a) SEM and (b) AFM images of GO flakes spin-coated onto a SiO_2/Si substrate. (c) Raman spectrum of GO.

Supplementary Information 2: CNTs crossing over a trench

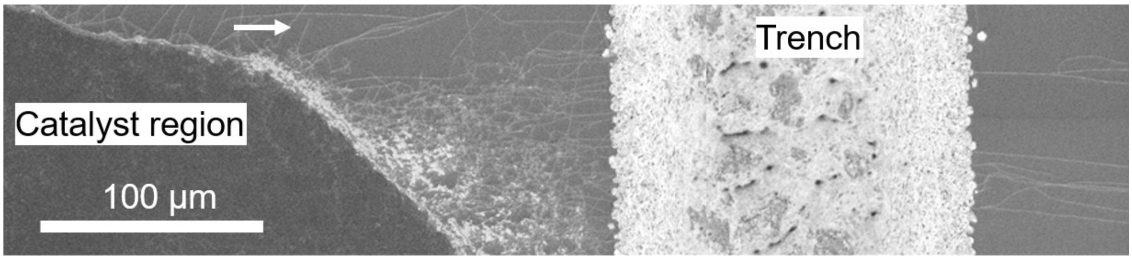

Figure S2. SEM image showing the growth of CNTs across a trench (width: ~100 µm, depth: ~63 µm). The arrow indicates the gas flow direction.

Supplementary Information 3: Definition and calculation of the delay time

"The delay time" in the manuscript (t_{delay}) was meant to be the time required for the sample to wait on the center of the furnace to contact with carbon source containing gas. Thus, except t_{delay} = 0 s, where CH_4 gas was continuously flowing the furnace when the catalyst substrate inserted into the furnace, the delay time was defined as $t_{delay} = t_{calib} + t$, where t is the timing of CH_4 gas valve opening from the moment the sample reaching the center of the furnace, and t_{calib} is time required for the CH_4 gas to reach the catalyst substrate from gas valve. t_{calib} was simply calculated as $t_{calib} = V / u$, where V is the volume of the gas flow path from the valve to the center of the furnace, and u is the gas flow rate. In this experimental setup, V was 170 cm^3 and u was 1000 cm^3/s. Although a part of V (~70 cm^3) was located in a heating zone of the furnace, expansion of the heated gas was neglected in the present calculation.

In the present experiment described in Figure 2, t of 0, 6, or 30 s was chosen, and thus the calculated t_{delay} was 10, 16, 40 s, respectively.

Supplementary Information 4: Fe_3O_4 particles supported on GO flakes

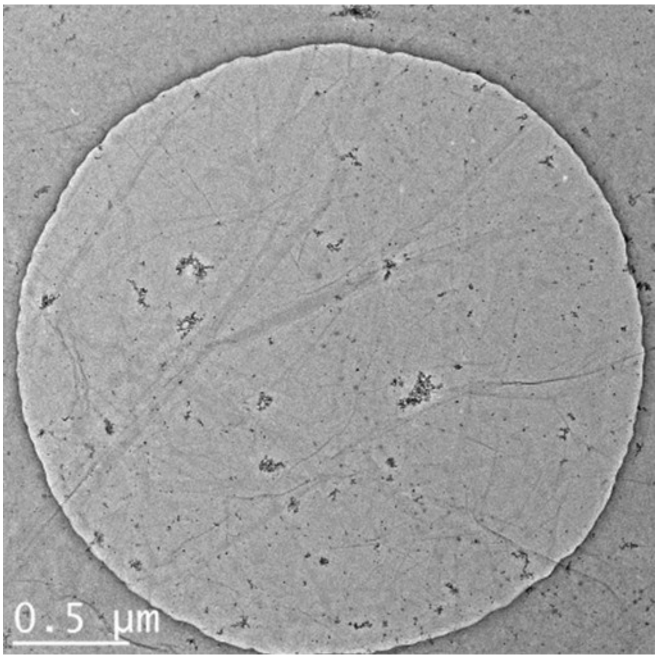

Figure S3. Low magnification TEM image of Fe_3O_4 particles supported on the GO layer covering a 2 µm microporous region of the silicon nitride membrane TEM grid.

Supplementary Information 5: Result of CNT growth using neither fast-heating method nor GO support

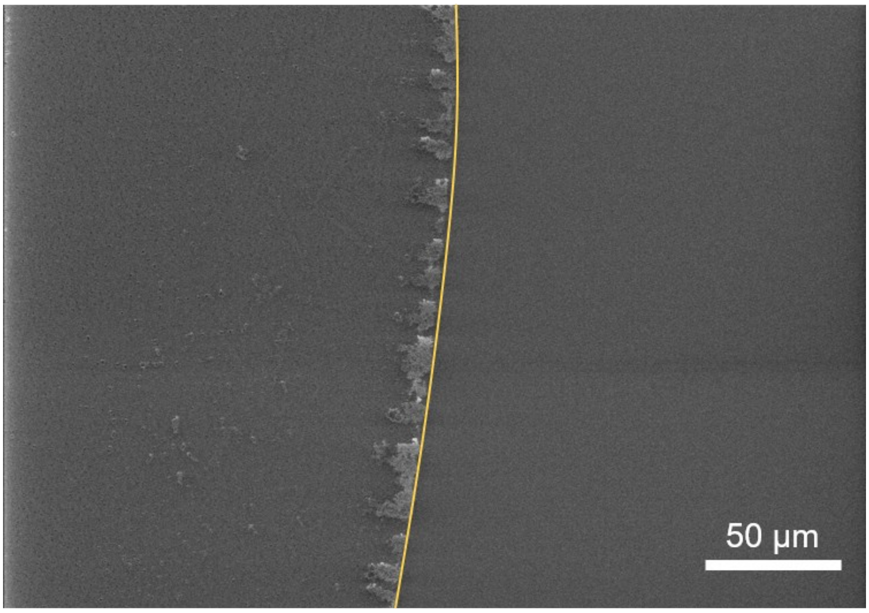

Figure S4. SEM image of the Fe_3O_4 catalyst coated SiO_2/Si substrate after the CVD process. In this CVD process, carbon source (CH_4) was supplied to the catalyst coated substrate after 40 second H_2 reduction process (delay time is 40 s). The area to the left side of yellow line is a catalyst region.

Supplementary Information 6: Effect of Fe_3O_4/GO ratio on the number density of grown CNTs

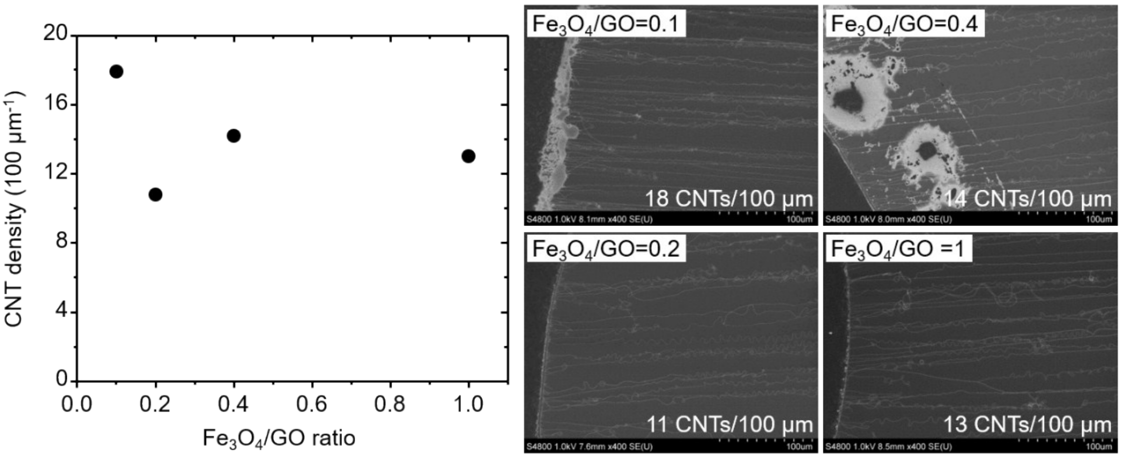

Figure S5 Number density of CNTs as a function of mass ratio of Fe_3O_4 against GO in the catalyst systems.
